# The Influence of Laser Defocusing in Selective Laser Melted IN 625

**DOI:** 10.3390/ma14133447

**Published:** 2021-06-22

**Authors:** Alexandru Paraschiv, Gheorghe Matache, Mihaela Raluca Condruz, Tiberius Florian Frigioescu, Ion Ionică

**Affiliations:** 1 Special Components for Gas Turbines Department, Romanian Research and Development Institute for Gas Turbines COMOTI, 220D Iuliu Maniu, 061126 Bucharest, Romania; gheorghe.matache@comoti.ro (G.M.); raluca.condruz@comoti.ro (M.R.C.); tiberius.frigioescu@comoti.ro (T.F.F.); 2 SC Plasma Jet SRL, 401E Atomiștilor, 077125 Magurele, Romania; officeplasmajet@gmail.com

**Keywords:** SLM, defocusing, IN 625, melt pool, tensile testing, density

## Abstract

Laser defocusing was investigated to assess the influence on the surface quality, melt pool shape, tensile properties, and densification of selective laser melted (SLMed) IN 625. Negative (−0.5 mm, −0.3 mm), positive (+0.3 mm, +0.5 mm), and 0 mm defocusing distances were used to produce specimens, while the other process parameters remained unchanged. The scanning electron microscopy (SEM) images of the melt pools generated by different defocusing amounts were used to assess the influence on the morphology and melt pool size. The mechanical properties were evaluated by tensile testing, and the bulk density of the parts was measured by Archimedes’ method. It was observed that the melt pool morphology and melting mode are directly related to the defocusing distances. The melting height increases while the melting depth decreases from positive to negative defocusing. The use of negative defocusing distances generates the conduction melting mode of the SLMed IN 625, and the alloy (as-built) has the maximum density and ultimate tensile strength. Conversely, the use of positive distances generates keyhole mode melting accompanied by a decrease of density and mechanical strength due to the increase in porosity and is therefore not suitable for the SLM process.

## 1. Introduction

Selective laser melting (SLM) is one of the most promising laser powder bed fusion (LPBF) techniques that has gained increasing attention in the last decade because of its ability to produce customized and functional parts with a complex geometry that would be difficult or even impossible to produce with standard subtractive manufacturing technologies [1,2,3,4,5].

Nevertheless, the lack of a full understanding of the impact of all process parameters on the quality of SLMed parts is still a limitation of this technology. Over 50 process parameters are involved in the SLM process that must be optimized to obtain high-density parts with tailored microstructures and high strength [6,7,8]. The process parameters are considered globally critical in the SLM process in terms of melt pool characteristics (shape and size), mechanical properties, and density, and many studies have focused on optimizing them to maximize the potential of the SLM technology [4,8,9].

As in any pulsed laser technology (laser additive manufacturing, laser welding, laser-cladding process), the laser beam has a high impact on the final microstructure of the melt pool [4,10], weld seams [11,12], or coating layer, [13] and on the mechanical performance of SLMed parts [14], joints [12,15] or clad coatings [16].

The laser’s power in the SLM process is concentrated on a spot, allowing for complete melting of the powder layer and partial melting of the previously molten layer. The SLM machines use a small laser beam focus diameter of less than 100 µm when the laser has a power of up to 600 W and a larger laser beam focus diameter (100–500 µm) when the laser has a power of up to 1 kW. In both cases, there are several advantages and disadvantages. Using a small spot of laser beam generates a small melt pool size and is beneficial in terms of microstructure and mechanical properties but reduces the manufacturing productivity [1] and increases the temperature gradients and cooling rates of the material, which may lead to the development of high residual stresses [5,17], keyhole mode melting [11], distortion, balling, evaporation [17], and microstructural defects [1,5]. However, when a higher spot is used, the manufacturing productivity can be slightly increased by reducing the number of scan lines [9], but the part’s dimensional accuracy and surface roughness can be compromised [1].

The spot of the laser beam and, consequently, the energy density influence the laser-melting modes, known in the domain of laser welding as the “conduction mode” and “keyhole mode” [1,4,8,9,14,18,19,20]. In the conduction mode, the depth of the melt pool is controlled by thermal conduction [10] and results in a semicircular melt pool shape [4,20], while in the keyhole mode, the formation of the melt pool is controlled by the evaporation of material and its depth is higher than in the conduction mode [10].

Due to these particularities, the melting mode is identified based on the ratio between the depth and width of the melt pool [4]. Additionally, a transition threshold between the melting modes is considered to be between 10^5^ and 10^6^ W/cm^2^ [18].

Many studies have reported that the keyhole mode in the LPBF process is unsuitable [1,4,10,18,19]. Metelkova et al. [4] showed that the melt pool is stable during the conduction mode, but becomes deep with spherical voids during the keyhole mode. King et al. [10] found the conditions in terms of laser parameters (power, speed, and spot size of the laser) required to avoid the transition from conduction mode to keyhole mode for a SLMed 316L stainless steel.

The transition from keyhole mode to conductive mode, or vice versa, can be influenced using a negative or positive defocusing distance, as was shown in several studies [4,14,19]. The defocusing distance represents the depth of penetration of the laser beam into the powder layer up to a certain distance from the surface of the powder bed that can affect the solidification path, melt pool morphology, density, and mechanical properties of the SLMed alloy [4,8]. McLouth et al. [19] changed the focal heights of the laser at three values (−3, 0, and 3 mm) and found that the focal shift alters the power density and microstructure of SLMed IN 718. Leo et al. [14] found that the defocusing distance (−1, 0, and 1 mm) affects the sizes of the melt pool and grain of SLMed 17-4PH but does not affect the number of defects. However, the specimens built with 0- and 1-mm defocusing distance had the best properties in terms of tensile strength and hardness.

Besides optimising other process parameters in the SLM process, finding an appropriate defocusing distance for the manufacturing of a part with specific requirements in terms of quality surface, density, microstructure, and mechanical properties can be challenging.

As shown by McLouth et al. [19], despite the similarities between different additive manufacturing processes, the influence of the defocus on porosity and microstructure cannot be generalized among processes, not even for the same type of alloy.

In recent years, extensive studies have been done mainly on the influence of the process parameters, such as laser power, laser speed, layer thickness, hatch spacing, and scanning strategy, on the microstructure and properties of the alloys, but the laser defocus has not been investigated as thoroughly.

Recent studies were done on the laser defocus influence on the microstructure of the additive manufactured alloys IN 718 [19], 17-4PH, 316L steels [14], AlSi10Mg [8], and Ti-6Al-4V [21] but less on IN 625 produced by selective laser melting.

In order to advance the knowledge on the influence of process parameters on material properties, the present study aimed to assess the influence of negative and positive defocusing distances on the melt pool size and morphology, densification, and tensile properties of selective laser melted IN 625.

## 2. Materials and Methods

For this study, prismatic specimens for the microstructural analysis and densification and round tensile specimens were manufactured using a Lasertec 30 SLM machine (DMG MORI, Bielefeld, Germany) equipped with a 600W Yb: YAG fiber laser and IN 625 metal powder manufactured by LPW Technology Ltd. (Runcorn, UK) with the chemical composition presented in Table 1. The actual chemical composition of the metal powder was supplied by the manufacturer. The metal powder particle size distribution measured by the authors in a previous work was D10 = 22 µm, D50 = 34 µm, and D90 = 42 µm [22].

The specimens were built with no contour in a vertical position, using five defocusing distances (−0.5 mm, −0.3 mm, 0 mm, 0.3 mm, and 0.5 mm), while the following process parameters were kept constant: 250 W laser power, 750 mm/s laser speed, 40 µm layer thickness, 0.11 mm hatch distance, and scan pattern of 90° between two successive layers. In the case of the Lasertec 30 SLM machine, when a negative defocusing distance is set, the focal plane of the laser beam is located at a certain distance above the powder bed, while for a positive defocusing, the focal plane is moved into the depth of the powder bed. The size of the laser beam with different defocusing distances was measured using an Ophir-Optronics beam analyzer equipped with a charge-coupled device (CCD) camera and a commercial beam profiling software (BeamGage Professional, version 6.12, Ophir Optronics Solutions Ltd., Jerusalem, Israel).

The specimens were manufactured on a preheated building plate (80 °C), under argon flow to maintain 0.2% oxygen in the building chamber, using a cross-type support structure with the geometry, dimensions, and process parameters presented elsewhere [23].

The roughness of the top surface of the specimens manufactured with different defocusing distances was evaluated using a mobile roughness measuring instrument (MarSurf-PS-10, Mahr Inc., Providence, RI, USA).

**Figure 1:**
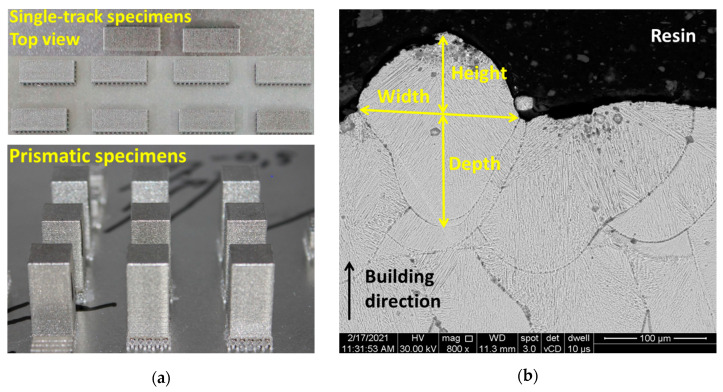
Prismatic specimens design used for the density measurements and melt pool analysis: (**a**) single-track and density specimens and (**b**) representation of the measured melt pool width, height, and depth of penetration on the cross section of single-track specimens.

The effects of defocusing distances on the melt pool depth, width, and height were analyzed by scanning electron microscopy (SEM) on sets of two specimens of 30 mm × 10 mm × 5 mm in size with a series of 50 single tracks manufactured on the last layer at a distance of 500 µm from each other (Figure 1a). Before the microstructural investigation, the single-track specimens were mounted in a resin (hot mounting) and fixed in the stainless steel clamping devices and T-slotted table of the abrasive disc-cutting machine. The specimens were cross sectioned, and the surface to be investigated was metallograpically prepared with sandpaper of varying grits (up to 1200) and polishing wheels with varying diamond suspension (3 and 1 microns) and etched with Aqua Regia reagent for 20 s. For each defocusing distance, 100 single tracks were analyzed in cross-section, transverse to the melting direction under the scanning electron microscope at 800× magnification. The melt pool dimensions were measured with the operating program of the microscope (XT microscope control 4.1.4.2010, FEI Company, Brno, Czech Republic).

The bulk density of sets of two prismatic specimens of 10 mm × 10 mm × 20 mm in size (Figure 1a) was measured via the Archimedes’ method using an analytical balance equipped with a density measurements kit with an accuracy of 0.0001 g. (Pioneer PX224, Ohaus Europe GmbH, Nänikon, Switzerland). The specimens were immersed in an auxiliary fluid (99.3% purity ethanol) with a known density variation with temperature according to the International Organization for Standardization (ISO) 3369:2006 [24] after the support material was removed by grinding with sandpaper. A theoretical density of 8.49 g/cm^3^ was calculated based on the actual chemical composition presented in Table 1 and was used as a reference to calculate the relative density of SLMed IN 625.

**Figure 2:**
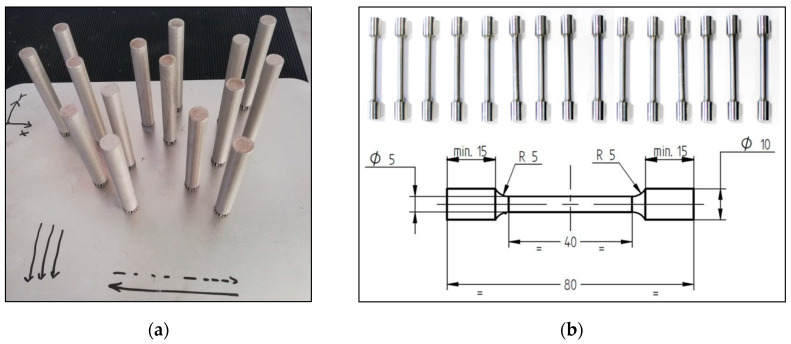
Tensile specimens: (**a**) cylindrical rods manufactured and (**b**) tensile specimen dimensions after machining (units in millimeters).

Sets of three cylindrical rods of 11 mm in diameter and 80 mm long (Figure 2a) were manufactured with respect to the building direction (Z) in the same conditions and using the same process parameters as the prismatic specimens to assess the influence of defocusing distances on the tensile properties of IN 625.

The cylindrical rods were subjected (prior machining) to heat treatment in air using an electric air furnace (Nabertherm LH 30/14 GmbH, Lilienthal/Bremen, Germany) that consists of stress relief heat treatment (heating with 10 °C/min up to 870 °C, held for 1 h, followed by air cooling) and annealing heat treatment (heating with 10 °C/min up to 1000 °C, held for 1 h, followed by fast cooling and oil quenching). The heat-treated rods were machined to obtain standard specimens (Figure 2b) and tested by using the Instron 3369 mechanical testing system (Instron, Norwood, MA, USA) according to ISO 6892-1:2009 [25]. The same heat treatment, geometry, and dimensions of the tensile specimens were used by the authors in other studies [23,26] to investigate the mechanical properties of SLMed IN 625.

## 3. Results

### 3.1. Macrostructural Analysis

**Figure 3:**
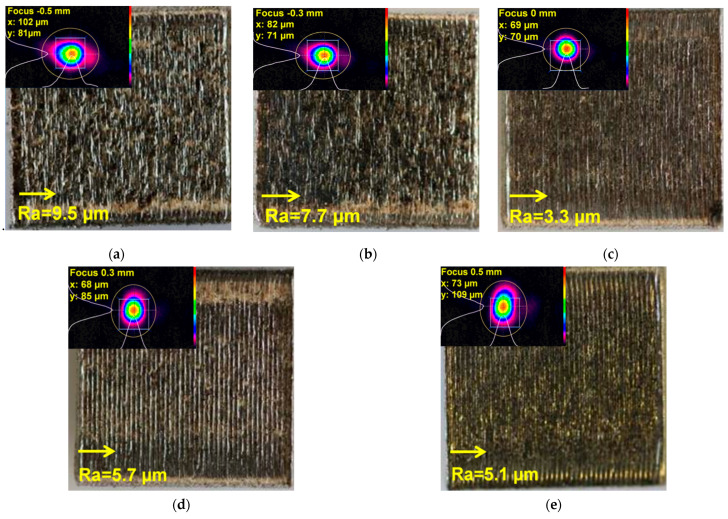
Macroscopic top-view images of the manufactured specimens built with different defocusing distances: (**a**) −0.5 mm, (**b**) −0.3 mm, (**c**) 0 mm, (**d**) 0.3 mm, and (**e**) 0.5 mm.

The spot size of the laser beam changes in the x- or y-axis with respect to negative or positive defocusing according to the 2D beam displays presented in Figure 3a–e, where the macroscopic top-view images of the manufactured specimens and the top-surface roughness value (Ra) are also presented.

As shown in Figure 3a–e, the change of defocusing distance from positive to negative had a high impact on the surface roughness and quality. The surface roughness was measured perpendicular to the scanning rotation (90°) for all specimens, and the lowest value (Ra = 3.3 µm) was obtained when the defocusing distance was set to 0 mm. Generally, increasing the laser beam diameter [4] or decreasing the offset focus [21] increases the surface roughness of parts manufactured by the LPBF process due to the flow instability induced by high-intensity laser irradiation that affects the melt pool behavior during the melting process.

### 3.2. Melt Pool Behavior

**Figure 4:**
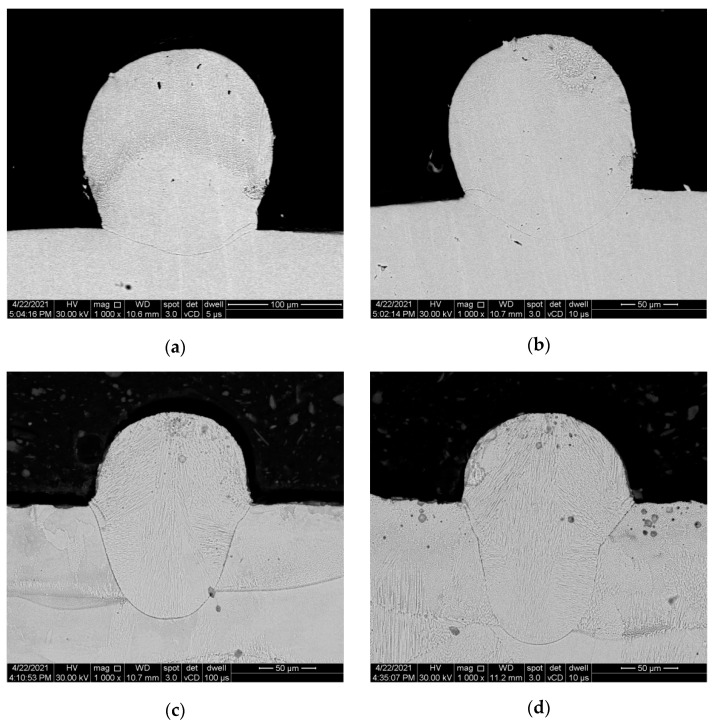

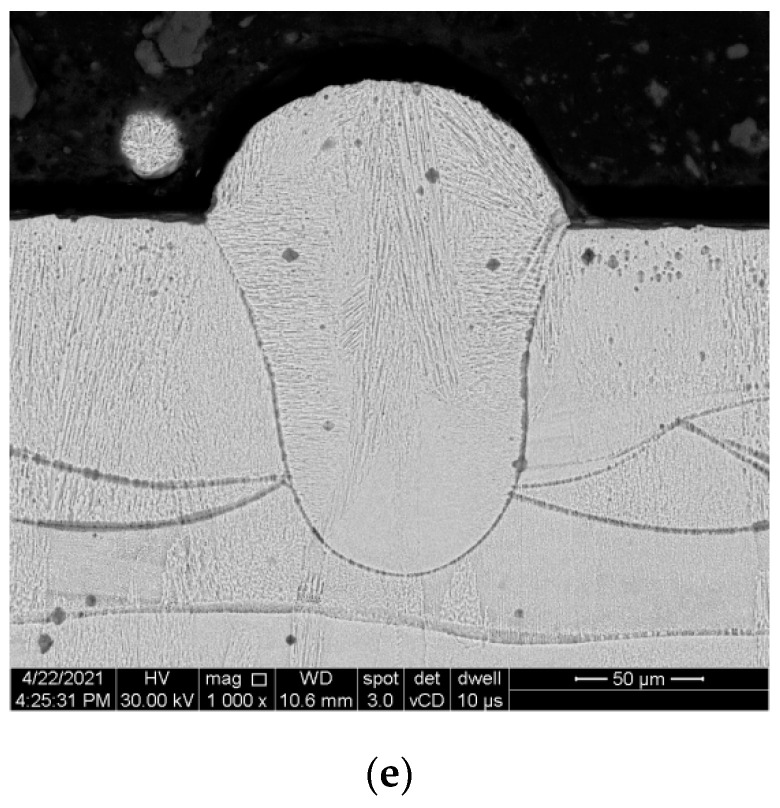
Cross-section SEM images of the single-track specimens built with different defocusing distance: (**a**) −0.5 mm, (**b**) −0.3 mm, (**c**) 0 mm, (**d**) 0.3 mm, and (**e**) 0.5 mm.

The SEM images of the cross-section of the single-track specimens built with defocusing distances of 0.5 mm, 0.3 mm, 0 mm, −0.3 mm, and −0.5 mm are presented in Figure 4a–e and were acquired to evaluate the influence of defocusing amounts on the dimensions and morphology of the melt pool.

As shown in the SEM images presented in Figure 4a–e, the defocusing distance substantially impacts the melt pool dimensions and morphology. As a general observation, the melting height increases while the melting depth decreases from positive to negative defocusing. The quantitative measurements of the melt pool depth, width, and height are presented in Table 2.

**Figure 5:**
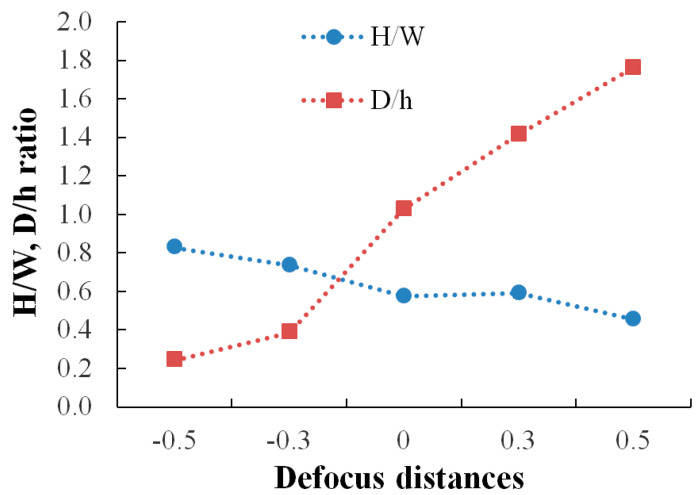
Influence of defocusing on melt pools height/width and depth/height ratio.

As shown in Figure 5, the height/width ratio decreases and the depth/height ratio increases with increasing the defocusing distance.

Based on the microstructural analyses of the melt pool behavior, it was found that even when using the same energy density, the melt pool became wider and less deep at negative defocusing distances (conduction mode) and narrower and deeper at positive defocusing distances (keyhole mode).

### 3.3. Density Measurements

The relative density of IN 625 specimens built with different defocusing distances was expressed as the ratio between the average density of specimens determined by Archimedes’ method and the theoretical density of IN 625 (8.49 g/cm^3^). The density of specimens was measured using an analytical balance equipped with a density measurement kit with an accuracy of 0.0001 g. The average values of density for each case (defocusing distance) were obtained based on 12 measurements with a slight standard deviation, as presented in Table 3.

**Figure 6:**
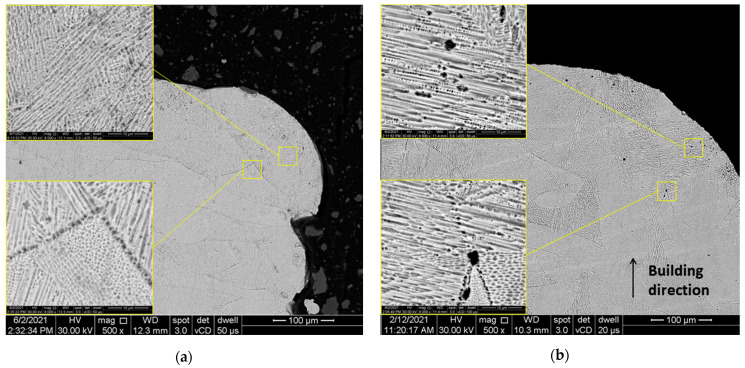
Porosity in SLMed IN 625: (**a**) with negative (conduction mode) and (**b**) positive (keyhole mode) defocusing distances.

Based on the density measurements presented in Table 3, it can be stated that the relative density of SLMed IN 625 with 0 mm and positive defocusing distances is maintained close to 99.3%, while at negative defocusing distances, the density slightly increases up to 99.5%. An explanation for this density evolution is the porosity that increases at positive values of the defocusing distances. Figure 6b highlights a higher porosity of the specimen built with a +0.5 mm defocusing distance than the specimen built with a −0.5 mm defocusing distance (Figure 6a). The micrographs shown in the windows present the details at higher magnification (8000×), highlighting porosities generated inherently by the melting and solidification processes. The differences between the pore size by defocusing shifting from negative to positive are visible.

### 3.4. Tensile Testing

Tensile tests on standard tensile test pieces machined from cylindrical rods built with different defocusing distances (−0.5 mm, −0.3 mm, 0 mm, +0.3 mm, and +0.5 mm) were performed at room temperature. The specimens were evaluated with respect to the ultimate tensile strength (UTS), 0.2% yield strength (YS), reduction of area (RA), and elongation (El).

The average tensile test results and standard deviations of each set of three specimens are presented in Table 4.

The SLMed alloys are very sensitive in terms of tensile properties due to the porosity, even in the case of up to 1% porosity, as was presented by Plessis et al. [27]. They conclude that the strength and ductility of LPBF materials are reduced with increasing porosity, and the failure is determined by the largest pores. The inherent presence of porosities and microcracks, because of the manufacturing method, act as stress concentrators, and therefore, fracture crack initiation and propagation under tensile load are influenced by their spatial distribution [28]. It was previously shown that cracks initiate on voids near the surface or in the subsurface and propagate radially [29].

**Figure 7:**
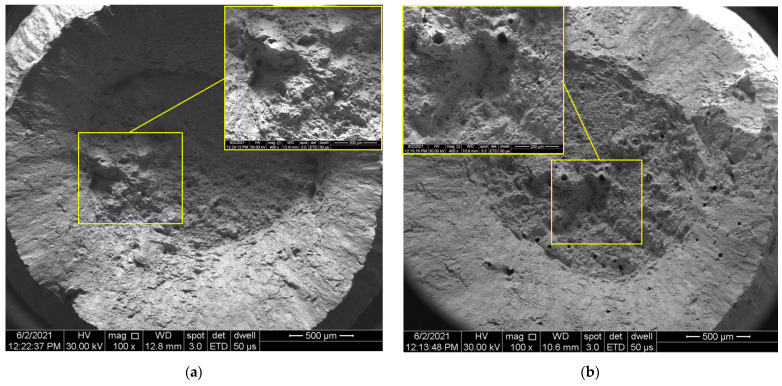

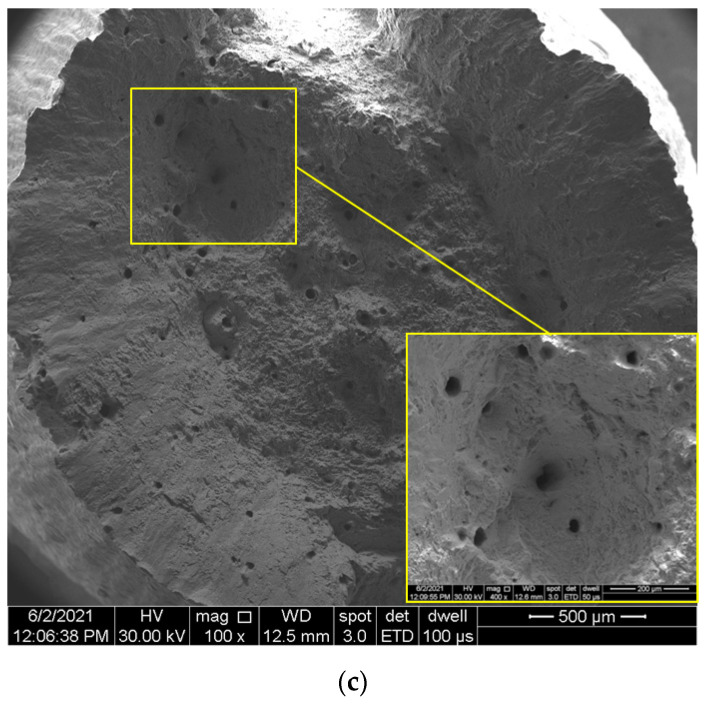
SEM images of the fracture surface of tensile test pieces built with different defocusing distances: (**a**) −0.5 mm, (**b**) 0 mm, and (**c**) +0.5 mm.

Figure 7 presents SEM images of the tensile fracture surfaces of test pieces built with negative (−0.5 mm), 0, and positive (+0.5 mm) defocusing distances. Micrographs in the windows present the details of the final fracture surfaces showing pre-existing porosities generated by the manufacturing process. Notably, it can be seen that positive defocusing is associated with a higher porosity level, which was also highlighted by relative density measurements.

The anisotropic behavior of SLMed IN 625 can also be related to the grain growth orientation over multiple layers (in the build direction) [30] and the sizes and morphology of the melt pool, which is strongly influenced by the defocusing distances. As was shown in Figure 4a–e, the melt pool became wider and thinner at negative defocusing. Therefore, the columnar grains grew on shorter distances and had lower mechanical strength than in the case of the 0 mm defocusing distances. However, at positive defocusing, the melt pool was deeper, and a higher mechanical strength would have been expected than in the other defocusing distances, but the higher porosity generated by the keyhole mode was a determining factor that weakened the ultimate tensile strength.

## 4. Discussion

The present study investigated the influence of laser defocusing on the surface quality, melt pool shape, densification, and tensile properties of selective laser melted IN 625.

When the defocusing distance was changed to positive or negative values, the melt pool was affected by the fluctuations from the thermocapillary convection and generated an increase of the surface roughness, especially in the case of negative distances, where the highest values were obtained (Ra = 7.7–9.5 µm).

During the solidification of the melt pool, the columnar dendrites become elongated in the growth direction normal to the solidification front [3], and the melt pool width and depth increase linearly with the increase of laser energy density [1]. These interactions between the laser beam and powder bed can generate a conduction mode, where the melt pool has a small depth and spherical shape, or a keyhole mode, where the depth of the melt pool is higher than half of its width [4]. The microstructural analysis revealed that the melting height and width decreases while the melting depth increases as the negative defocusing amount decreases. Another study [31] found the opposite effect of using negative defocusing distances, which can be explained by the beam divergence that can be reversed from one SLM system to another. The melting mode, and consequently the melt pool morphology and size, can also be influenced by a high laser power, low beam size, and low scanning speed [4,10]. However, the semicircular melt pool is obtained in the conduction mode (negative distances) and becomes narrower and deeper when the heat flow has a keyhole mode (positive distances) [4,10,14].

When varying the defocusing distances from negative distances to positive distances, the relative density of IN 625 prismatic specimens decreased from 99.52% (conduction mode) to 99.28% because of the increasing of the porosity, which usually occurs in the keyhole mode melting due to the vaporization of the overheated material [8,10,32]. Another effect of this variation is the weakening of the tensile properties. Both ultimate tensile strength (UTS) and yield strength 0.2% (YS) tend to slightly decrease with the variation of defocusing distance from negative to positive values, but no more than 2%.

Comparative experimental data obtained on SLMed IN 625 have not been identified in the literature. A similar variation was observed in another study by Zhou et al. [8] on a SLMed AlSi10Mg alloy. They found that the defocusing distances affected both the tensile mechanical properties and density of the SLMed alloy, and the highest relative densities and tensile properties were achieved in the conduction mode melting. However, in the study done by Leo et al. [14], the highest tensile strength of SLMed 17-4PH was obtained for the positive, with 0 and 1 mm defocusing distances.

The anisotropy of SLMed materials is well known, and many studies have demonstrated the influence of grain morphology, texture, building orientation, and scanning strategy on the anisotropic microstructure and tensile properties of SLMed Ni-based superalloys [5,26,33].

**Figure 8:**
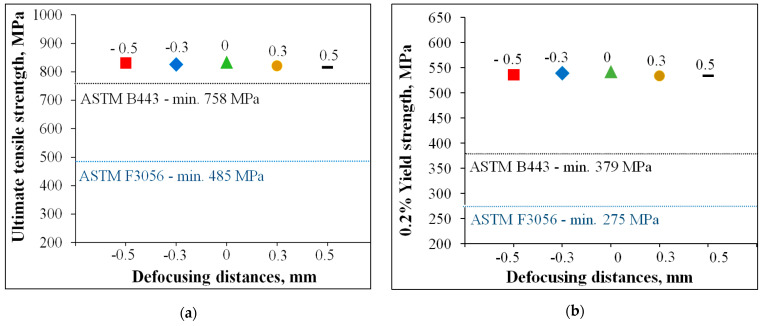

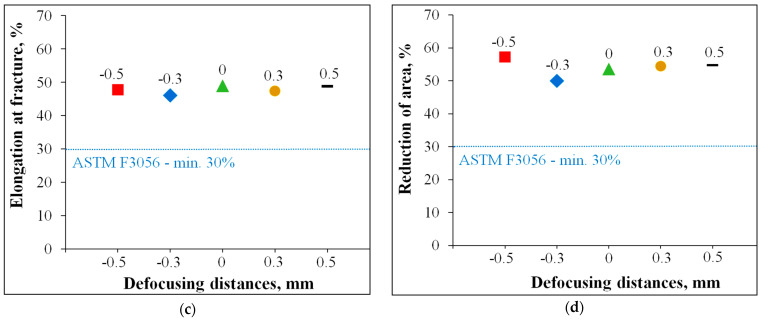
Tensile properties of specimens built with different defocusing distances: (**a**) UTS; (**b**) YS; (**c**) El; (**d**) RA.

However, the tensile properties of IN 625 specimens built along the Z-axis with different defocusing distances exceed the minimum specification requirements for both conventional and additively manufactured IN 625 alloys according to the American Society for Testing and Materials, ASTM B 443 [34] and ASTM F3056-14e1 [35], respectively, as is presented in Figure 8. The tensile results presented in Table 4 are also presented in a graphic form in Figure 8 to compare them with the minimum requirements of the ultimate tensile strength and yield strength of additively manufactured IN 625 and hot-rolled IN 625.

Figure 8a–d presents the tensile test results of specimens built with different defocusing distances, where the dashed lines represent the minimum values for additively manufactured IN 625 according to ASTM F3056-14e1 [35]. Additional dashed lines were also plotted for the hot-rolled IN 625 plates according to ASTM B 443 [34]. In addition to the higher strength values of SLMed specimens relative to the minimum values, the elongation and reduction of area after fracture are higher than the minimum values of both ASTM, which shows a higher ductility due to the orientation of the columnar grains relative to the loading direction.

## 5. Conclusions

Laser defocusing was investigated to assess the influence of negative (−0.5 mm, −0.3 mm), 0 mm, and positive (−0.3 mm, 0.5 mm) defocusing distances on the melt pool width, height, and depth of penetration, surface roughness, densification, and tensile properties of selective laser melted IN 625.

The defocusing distances had a high impact on the quality of the surface. The lowest surface roughness (Ra = 3.3 µm) was generated when the defocusing distance was set to 0 mm but increased from positive to negative defocusing distances up to Ra = 9.5 µm (for −0.5 mm defocusing distance).

The use of negative defocusing distances generates a conduction melting mode of the SLMed IN 625, where the melt pool has a small depth and spherical shape. Conversely, the use of positive distances generates a keyhole mode melting, where the depth of the melt pool is higher than half of its width. As a general trend, the melt pools tended to become wider and thinner at negative defocusing (conduction mode) and narrower and deeper at positive defocusing distances (keyhole mode).

When negative defocusing amounts (−0.3 and −0.5 mm) were used, a relative density higher than 99.5% was obtained, while in the cases of 0 mm and positive defocusing distances (0.3 and 0.5 mm), the relative density was slightly reduced close to 99.3%.

The tensile test results showed that the defocusing distances slightly influenced the tensile properties of IN 625. Both ultimate tensile strength (UTS) and yield strength (YS) tend to decrease very slightly with the variation of defocusing distance from negative to positive values due to the loading direction, the orientation of the columnar grains grown over multiple layers, and the anisotropy of the SLMed IN 625.

## Figures and Tables

**Table 1:** Chemical composition of IN 625.

Elements (%wt.)	Al	C	Co	Cr	Fe	Mn	Mo	Nb	Si	Ti	Ni
Specification	<0.4	<0.1	<1.0	20–23	3–5	<0.5	8–10	3.15–4.15	<0.5	<0.4	Bal.
Actual composition	0.06	0.02	0.1	20.7	4.1	0.01	8.9	3.77	0.01	0.07	62.26

**Table 2:** Melt pools size for different defocusing amounts.

Defocusing, mm	−0.5	−0.3	0	0.3	0.5
Width, µm	151 ± 32	146 ± 26	150 ± 17	137 ± 12	143 ± 10
Depth, µm	30 ± 11	42 ± 16	89 ± 23	114 ± 21	115 ± 22
Height, µm	125 ± 40	107 ± 33	86 ± 33	80 ± 17	65 ± 16

**Table 3:** The relative density of SLMed IN 625 as a function of defocusing amounts.

Defocusing, mm	−0.5	−0.3	0	0.3	0.5
Relative density, %	99.52	99.52	99.37	99.27	99.28
Standard deviation, %	0.02	0.03	0.02	0.09	0.06

**Table 4:** Tensile properties of specimens built with different defocusing distances.

Tensile Properties	−0.5	−0.3	0	0.3	0.5
Ultimate tensile strength, MPa	831 ± 3.3	826 ± 5.7	834 ± 6.4	821 ± 3.9	816 ± 4.1
0.2% Yield strength, MPa	536 ± 3.3	539 ± 3.3	542 ± 5.4	534 ± 5	532 ± 4
Reduction of area, %	57 ± 1	50 ± 2.6	54 ± 1.9	54 ± 1.6	55 ± 0.5
Elongation, %	48 ± 0.8	46 ± 1	49 ± 1	47 ± 0.3	49 ± 0.3

## Data Availability

Data sharing not applicable.
